# Organs-on-chip: The way forward

**DOI:** 10.1016/j.stemcr.2021.06.015

**Published:** 2021-07-22

**Authors:** Massimo Mastrangeli, Janny van den Eijnden-van Raaij

**Affiliations:** 1 Delft University of Technology, Delft, the Netherlands; 2 Human Organ and Disease Model Technologies (hDMT), the Netherlands

**Keywords:** organ-on-chip, microphysiological systems, stem cell, standardization, qualification, roadmap, drug development, disease modeling, personalized medicine

## Abstract

Organ-on-chip (OoC) technology is thriving thanks to stem cells availability and international OoC programs. Concerted standardization, qualification, and independent testing of devices are needed to coherently develop OoC technology further and fulfill its potential in drug development, disease modeling, and personalized medicine. The OoC roadmap can lead the way forward.

## Teaser

In this article, Mastrangeli and van den Eijnden-van Raaij offer a perspective on the way forward for organ-on-chip technology, emphasizing the need for concerted efforts in standardization, qualification, and independent testing of devices for a coherent development of this thriving technology and fulfillment of its potential. The way forward is laid out in a roadmap.

## Main text

### Organ-on-chip: Technology and roadmap

Organ-on-chip (OoC) is an emerging technology that benefits from the convergence of stem cells and tissue engineering with microfluidics and microfabrication of sensors and actuators. OoC models aim to recapitulate aspects of human physiology and pathology as improvements to existing bioassays, and to provide insights into mechanisms underlying drug responses and development and progression of disease. Mounting evidence indicates that OoC devices (OoCs) may provide better model systems for research on health and disease. The evidence includes showcases of vessels-on-chip, cancer-on-chip, kidney-on-chip, neurons and glia cells-on-chip, lung-on-chip, and ALS-on-chip (Mastrangeli et al., 2019b). Utility of OoC models is already foreseen in drug discovery, efficacy, and toxicology, and, with the advent of stem cells derived from patients, in precision or even personalized medicine. OoC based on human cells might also reduce the need, cost, and ethical burden of animal studies.

Although the OoC field is still in its infancy, OoC models are being widely developed by academia and industry, increasingly based on adult or human induced pluripotent stem cells (hiPSCs), primary human cells and cell lines, or organoids. The models range from those representing single-organ systems to multi-organ- and even body-on-chip formats (Marx et al., 2016, 2020; Park et al., 2019; Low et al., 2020; Picollet-D’Hahan et al., 2021). Some models are already being used to gain insight into disease etiology and identify drug target pathways. Moreover, a number of models have appeared as “low-hanging fruit” and show evidence of representing better alternatives to certain animal models of reference. However, many OoCs are not yet robust for all cell types, are not reproducible from experiment to experiment or user to user, and should moreover be independently qualified as fit for purpose. In addition, they are not always compatible with existing lab workflows of end users. These and other hurdles remain to be addressed to realize OoC adoption by industry and acceptance of OoC models by regulators as animal alternatives.

**Figure 1:**
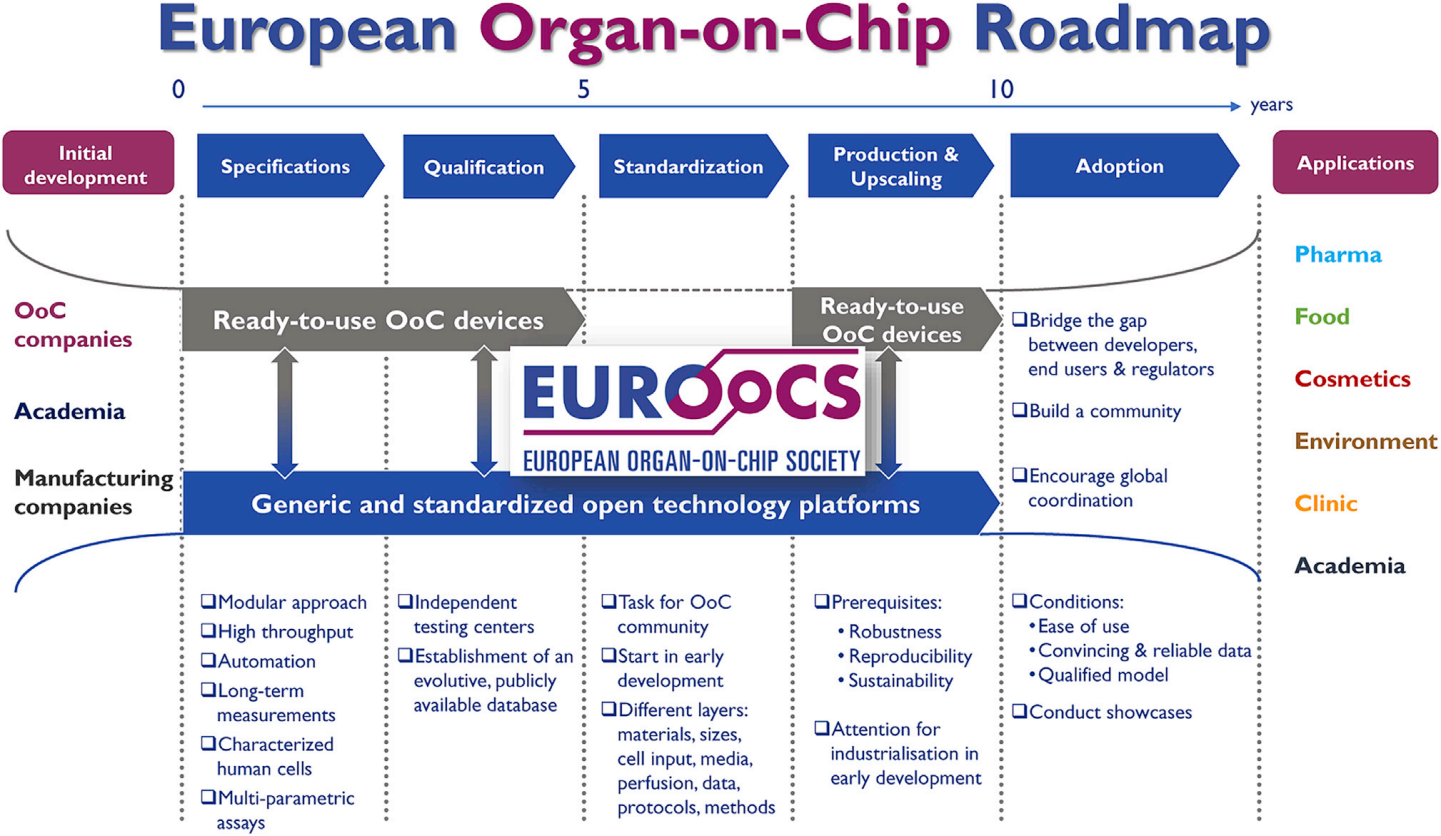
The European OoC roadmap From Mastrangeli et al. (2019b) under Creative Commons License.

An inventory of the unmet needs, key challenges, barriers, and perspectives of OoC technology was recently published as the outcome of the Horizon 2020 FET-Open project “Organ-on-Chip In Development” (ORCHID) (Mastrangeli et al., 2019a). Similarly, a recent workshop of the Transatlantic Think Tank for Toxicology (T4) identified major challenges for the adoption of OoC technology—which included lack of standardization and qualification for specific purposes, and limited communication between different stakeholders—and proposed potential solutions (Marx et al., 2020). These insights and the active involvement of more than 70 world-renowned experts—including developers, end users, and regulators—led to the development of the European OoC roadmap (Figure 1; Mastrangeli et al., 2019b). A second outcome of ORCHID was founding of the European Organ-on-Chip Society (EUROoCS) as key supervising entity for the implementation of the roadmap. EUROoCS is now affiliated with the International Society for Stem Cell Research with remit to review and co-publish combined OoC-stem cell research in *Stem Cell Reports*. As described below, several current and future national, European, and global OoC initiatives will take the first important steps described in the roadmap toward wide OoC implementation and training the next generation of OoC researchers.

### First steps along the roadmap: Standardization and qualification

#### Standardization

Standardization of OoCs is challenging, since OoC technology is inherently interdisciplinary. Multiple approaches and devices have already been proposed to meet user and application needs that require both biological and engineering expertise. The dilemma for many is which OoC is the best for the questions to be addressed. On the one hand, variety promotes the development of effective solutions and aligns with evidence that a single OoC device is unlikely to fit all purposes. On the other hand, diversification runs the risk of fragmenting the OoC landscape such that there are multiple “single-issue” solutions with limited research or commercial availability once a project has finished. Although adoption of standards and guidelines in microfluidics to address this has been slow (Reyes et al., 2021), successful examples of standardization in the electronic components and systems (ECS) domain (e.g., data communication protocols, interfaces, and peripheral cross-compatibility) attest to the strategic advantages that timely and harmonized standardization can bring to a technological field.

**Figure 2:**
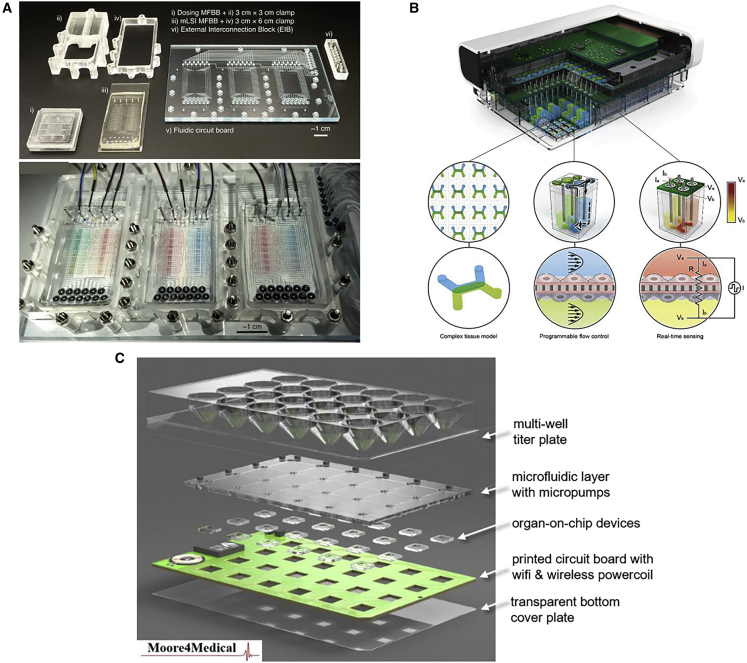
Examples of OoC platforms (A) Fabricated parts (upper panel) and full assembly (lower panel) of the Translational OoC Platform (TOP). Adapted from Vollertsen et al. (2020) under Creative Commons License. (B) Predict96, the high-throughput OoC platform with integrated pumping and sensing from Draper. Adapted from Azizgolshani et al. (2021), published by The Royal Society of Chemistry. (C) The four functional layers of the Moore4Medical's Smart Multi-Well Plate (SMWP) platform (an animated illustration of the SMWP concept and architecture is available at https://youtu.be/H595oGbMdyM). Credit: Moore4Medical ECSEL JU.

Standardization of OoC entails a hierarchy of layers, ranging from the materials used (substrates, cells, perfusion media), the devices themselves (size, footprint, functionality, accessibility), interfaces (cross-compatibility, back-compatibility with existing laboratory instrumentation and workflows), and assays (cell handling, phenotypic and genotypic characterization, cell differentiation protocols, endpoints) to the actual data (formatting, analysis, archiving, sharing). The establishment of open technology platforms was recommended in the OoC roadmap as the solution to gathering knowledge and expertise from users and developers and, correspondingly, immediately addressing at a systems level the need for OoC devices which are standardized, easy to use, and compatible with existing laboratory practice. As a technology platform, OoCs should provide end users with the possibility to select devices that best fit their purpose from a set of available technological options. There is a multiplicity of OoCs commercially available. These include devices from Hesperos (https://hesperosinc.com/), Aim Biotech (https://aimbiotech.com/), InSphero (https://insphero.com/), Nortis (https://nortisbio.com/), and Emulate (https://www.emulatebio.com/). Examples of complete, multi-chip platforms include the commercially available devices from Mimetas (https://www.mimetas.com/en/home/) and TissUse (https://www.tissuse.com/en/). These commercial devices have different sizes and formats, and cannot be mutually linked with ease. To overcome such lack of standardization in forms and interfaces, several platforms for OoC are currently being developed, including the Translational OoC Platform (TOP) developed at the University of Twente (Vollertsen et al., 2020), the Predict96 high-throughput OoC platform from Draper (Azizgolshani et al., 2021), and the smart multi-well plate (SMWP) under development in the Electronic Components and Systems for European Leadership Joint Undertaking (ECSEL JU) project “Moore4Medical”. We briefly review these platforms below (Figure 2).

TOP is a modular microfluidic platform whereby an ISO-standardized fluidic circuit board (FCB) is used to drive plug-and-play microfluidic building blocks (MFBBs) (Figure 2A). One FCB can fully automatically link and operate up to three MFBBs. The MFBBs can be the same or have different designs and functions (e.g., with respect to cell culture and sensing). Fluidic multiplexing is controlled in both the FCB and MFBBs through integrated polymeric valves. TOP can control MFBBs with 64 microchambers and support long-term cell culture with high spatiotemporal control, as well as allow liquid dosing with high dynamic range. Ongoing research aims to investigate whether TOP can accommodate OoC systems and/or MFBBs from different providers. The ultimate goal is to set a standard for OoCs analogous to the 96- or 364-well plates for screening in standard cell cultures.

Draper's platform is composed of 96 individual OoC devices in a standard 96-well plate that is compatible with high-content screening tools, a perfusion system contained within a plate lid including up to 192 active micropumps, and a total of 384 electrical contacts embedded in the perfusion systems for real-time transepithelial electrical resistance measurements within each OoC well (Figure 2B). Each OoC device contains two microchannels separated by a semi-permeable scaffold, able to support a range of tissue types (e.g., liver, kidney, intestine, vascular). Fluid flow in each individual channel is controlled by a separate micropump. The platform employs optically clear thermoplastic materials that minimize drug absorption and enable histological analysis using standard microscopy techniques.

The SMWP was conceived as a standardized and stand-alone multi-well plate for the full automation of OoC assays and workflows. The SMWP features four main functional layers (Figure 2C): an open-top microplate with standard footprint; a user-configurable OoC layer, whereby OoCs, produced by different providers and embedding electrodes and sensors, have a standardized footprint and standardized electrical- and fluidic interfaces; a microfluidic distribution layer, embedding the OoCs within microfluidic circuits driven by integrated piezoelectric micropumps; and an electric distribution layer in the form of a printed circuit board, driving on-chip sensors and electrode readout, and hosting wireless modules for power and data transfer. The layers are housed within a hard plastic, optically transparent, and hermetically closed casing for use in standard cell culture incubator environments for advanced OoC biological assays.

Access, quality control, and handling of human (stem) cell sources represent essential technical aspects of OoCs that need standardization. This is the reason to engage the stem cell community in relevant dialog. Each of the multiple cell sources currently available (i.e., primary adult human cells, adult stem cells, human embryonic stem cells, and hiPSCs, from both spheroids and organoids) have specific advantages and disadvantages. Important are uniformity and standardization in rigor, oversight, transparency, and ethical integrity in sourcing and handling of cells in accordance with recommended guidelines. The *Guidelines for Stem Cell Research and Clinical Translation* (https://www.isscr.org/policy/guidelines-for-stem-cell-research-and-clinical-translation) developed by the ISSCR, updated in May 2021, are the most prominent example, and are expected to be adopted by researchers and clinicians worldwide. More generally, the Organization for Economic Cooperation and Development (OECD) has laid the basis for internationally recognized test guidelines, implemented within a Good Laboratory Practice quality system (such as the OECD's *Guidance Document on Good In Vitro Method Practices* (https://www.oecd-ilibrary.org/environment/guidance-document-on-good-in-vitro-method-practices-givimp_9789264304796-en), and satisfying the conditions of Mutual Acceptance of Data between jurisdictions and regulatory agencies. Another instance is the Comprehensive In Vitro Proarrhythmia Assay initiative (https://cipaproject.org/), which aims to improve accuracy in predicting cardiac risk of drugs through their most prominent feature, namely their risk of causing sudden cardiac death. The validation processes foreseen in this context will be appropriate for highly standardized and widely applicable methods.

#### Qualification

Wide adoption of OoC models by end users and regulators has been hampered by the lack of information on the reliability, relevance, and added value of OoC technology. Characterization and qualification of OoC devices is therefore urgently needed to provide end users—from pharmaceutical and biotechnological industries to regulatory agencies—with confidence in the robustness of the data obtained. For drug screening and development, the characterization and qualification of OoCs should comply with key conditions: (1) defined context of use and associated outcomes to select the most relevant OoC model; (2) convincing outcomes of studies with reference compounds insofar as they have been classified regarding context of use and specific parameters; (3) robust quality control assays ensuring the functional characterization of (stem) cell cultures, material qualification (drug-biomaterial interaction), manufacturability, and availability of devices; (4) demonstrated effectiveness compared with current *in vivo* profiles; (5) intra- and inter-laboratory assays that demonstrate the reproducibility and accuracy of the OoCs as well as monitoring technological performance (stability and robustness).

Ideally, and as proposed by the US testing center initiative funded by the National Center for Advancing Translational Sciences, all qualification studies should be performed by a third party to ensure an independent analytical characterization. The Tissue Chip Testing Centers at Massachusetts Institute of Technology and Texas A&M University are carrying out independent experiments with a diverse range of models, and the data are deposited in a database developed by the University of Pittsburgh.

Design and implementation of a European multi-center OoC infrastructure for independent testing and qualification of OoC devices, data centers, and training of next-generation researchers is advancing with the support of organizations such as the EU Reference Laboratory for Alternatives to Animal Testing and EUROoCS. Pharma and regulatory bodies would ideally join this infrastructure initiative, which will be aligned with other large European research infrastructures. A publicly accessible database for storage of qualification data will be necessary to promote OoC adoption supported by early engagement of academic, industrial, and regulatory players. The coordination of the database with other international databases, like that in the United States mentioned above, should reinforce multi-partner task forces and contribute to international harmonization.

There are in addition national initiatives for setting up expertise centers which might serve as future testing centers. In the context of the hDMT (the Dutch OoC consortium), the Organ-on-Chip Center Twente and the iPS&OoC Hotel at the Leiden University Medical Center have been established as pilot centers to facilitate academic and industrial researchers in implementing OoC models. These centers are being developed further and serve as a blueprint for other expertise centers in the Netherlands. Their aim is to facilitate and structure OoC knowledge utilization. Ongoing research activities in the pilot centers and benchmarking studies (e.g., “showcases” using hiPSC derivatives in OoCs that exemplify utility beyond present gold standard models) will provide a strong basis for developing standardization and qualification for different types of models using clinically known reference compounds. Lists of reference compounds have already been developed for heart models (de Korte et al., 2020).

Collaboration between regulators, developers, and end users is crucial for the OoC qualification process. Together they should define the parameters for qualification of an OoC model for a specific context of use. Regulators, including the European Medicines Agency and the US Food and Drug Association (FDA) are already actively involved in this. They are keen to look for possibilities to accelerate adoption of OoC devices, in particular for those models that fill regulatory gaps. End users, united in the International Consortium for Innovation and Quality in Pharmaceutical Development (IQ Consortium, a not-for-profit organization of pharmaceutical and biotechnology companies), collaborate with the FDA and the National Institutes of Health in the United States on the qualification of OoC systems as *in vitro* tools for drug development. The IQ Consortium recently published a series of articles on the characterization and use of different OoC systems in safety and toxicity profiling applications (Fabre et al., 2020). They have compiled a list of reference compounds that might be shared for qualification purposes. This will be of great benefit for both users and developers.

### Production and upscaling

Substantial growth and wide implementation of OoC technology is expected to require an industrial upscaling of production volumes. Upscaling inherently involves both technological and biological components, as it implies mass production of reproducible devices and platforms as well as generation of large batches of differentiated and quality-controlled cells. In an ideal world, OoC plates prefilled with quality-controlled stem cell derivatives would arrive cryopreserved at the end-user site ready for use simply after thaw. In this regard, straightforward transfer from lab to lab and from lab to fab (e.g., foundry) represents an essential requirement for every technology eligible for upscaling, and it needs relevant choices of materials and fabrication processes with which to start. Moreover, standardization and qualification efforts mentioned above should ideally constrain variety and variability that may hinder biotechnological reproducibility. This would set the stage for large investments, which could be proposed in the context of public-private funding calls.

### EUROoCS: Connecting the stakeholders

It is clear that for convergence of component standardization, methods, and data, dialog among experts is required. To ensure such dialog is one goal of EUROoCS. A recent PSIS (Putting Science into Standards) workshop, jointly organized by the European Committee for Standardization, the European Committee for Electrotechnical Standardization, and the Joint Research Center, particularly recommended a bridging role for EUROoCS for standardization in the OoC roadmap. EUROoCS′ Regulatory Advisory Board and Industrial Advisory Board are supporting this within EUROoCS, together with the European standardization organizations and the European Commission. In addition, for OoC qualification EUROoCS is already encouraging the OoC community to come up with well-documented showcases and will catalyze the development of the qualification methodology and infrastructure for OoC. The expanding nationwide OoC networks—which are established or being set up in many countries including France, Israel, Spain, Switzerland, Scandinavian countries, the Netherlands, and the United Kingdom—will become the pillars of the EUROoCS community, which is currently developing the strategy from the bottom up with the requirements for implementation of the OoC roadmap.

### Next-generation organ-on-chip researchers

Many of the points advocated above to promote and advance the OoC field need community endeavors. As mentioned, involvement of all stakeholders in proactive communication, interaction, and sharing of data and expertise at all stages of the OoC roadmap is thereby central. This needs to be accompanied by dissemination of knowledge, results, and perspectives to wide and varied audiences and, not least, by creating a new cohort of researchers, equally knowledgable in biology and engineering. This would reflect the entwinement of engineering and biology that is at the heart of OoC, whereby the former provides solutions and constraints to the questions and targets set by the latter.

This type of hybrid education starts from the appreciation of a shared technical language, which goes beyond compartmentalization to fund the basis for transparency, mutual understanding, and matching of expectations, avoiding unnecessary blocks and misalignments. An exemplary initiative in this regard is the interdisciplinary training network for advancing OoC technology in Europe (EUROoC). EUROoC merges doctoral students with different backgrounds and at similar educational stages in formal and informal contexts to share expertise and knowledge. This is remarkably effective for “teaching by peers” through hands-on training, courses, seminars, collaboration, publications, and dissemination. Similar initiatives elsewhere will undoubtedly inspire undergraduate-level programs as well, so that OoC technology will become firmly embedded in the academic and applied curricula and engage many students to become next-generation OoC researchers.
